# Clinical predictors of short-term treatment response in stupor: a retrospective study

**DOI:** 10.3389/fpsyt.2025.1714338

**Published:** 2026-01-23

**Authors:** Xin Zong, Zhiqian Liu, Zhimeng Yang, Chenchen Zhang, Rui Li, Kun Li, Baohua Li

**Affiliations:** 1 Shandong Daizhuang Hospital, Jining, Shandong, China; 2 School of Mental Health, Jining Medical University, Jining, Shandong, China; 3 Jining Key Laboratory of Neuromodulation, Jining, Shandong, China

**Keywords:** efficacy, electroconvulsive therapy, inflammatorymarkers, olanzapine, predictors, stupor

## Abstract

**Background:**

Stupor is a severe psychomotor syndrome with motor retardation and speech inhibition, that adversely affects outcomes. Benzodiazepines and electroconvulsive therapy (ECT) are standard treatments, yet individual responses vary considerably, highlighting the need for effective clinical predictors.

**Objective:**

This study aimed to identify clinical predictors of therapeutic response in patients with stupor to facilitate individualized treatment.

**Methods:**

We conducted a retrospective observational study including 45 patients with stupor hospitalized at Shandong Daizhuang Hospital between January 2010 and August 2024. Finally, 40 patients met the inclusion criteria and were retained for analysis. Patients were classified as responders or non-responders based on Clinical Global Impression-Improvement (CGI-I) scores at two weeks. Demographic characteristics, clinical variables, and medication regimens, including antipsychotics, benzodiazepines, and ECT use, were extracted from medical records. Peripheral blood inflammatory markers were also collected and analyzed. Mann-Whitney *U* tests, chi-square tests, logistic regression analyses, and receiver operating characteristic (ROC) curves were used to identify predictive factors and evaluate their diagnostic accuracy.

**Results:**

Among 40 patients, 24 (60.00%) responded favorably. Responders were more likely to receive ECT (*χ^2^* = 6.667, *P* = 0.010), higher chlorpromazine equivalent doses (*|Z|* = 3.418, *P* = 0.001), and olanzapine (*χ^2^* = 4.365, *P* = 0.037), while inflammatory markers showed no differences. Logistic regression identified chlorpromazine equivalent dose as an independent predictor (*OR* = 1.007, 95% *CI*: 0.998-1.017, *P* = 0.045). ROC analysis suggested a data-driven, exploratory optimal predictive chlorpromazine-equivalent dose of approximately 108 mg (about 100–110 mg) (AUC = 0.820, 95% *CI*: 0.677-0.963), corresponding to roughly 4.3-5.4 mg of olanzapine.

**Conclusion:**

A higher chlorpromazine-equivalent dose and olanzapine use were associated with short-term therapeutic response in stupor, independent of ECT. ROC analysis suggested a data-driven, exploratory cut-off of approximately 108 mg (about 100–110 mg) chlorpromazine-equivalent, which should not be interpreted as a prescriptive clinical threshold. However, these findings are preliminary, limited to two-week outcomes in a modest retrospective sample. The role of inflammation in stupor remains inconclusive, and further studies are warranted to clarify its predictive value.

## 1 Introduction

Stupor is a severe psychomotor syndrome frequently encountered in psychiatric practice, typically characterized by profound motor retardation, mutism, and markedly reduced responsiveness (1). It occurs in various conditions, including schizophrenia, mood disorders, dissociative disorders, and organic brain diseases (2). Neurological, metabolic, immune, and pharmacological factors may also contribute to its etiology (3, 4). Epidemiological studies suggest that stupor is most commonly observed in patients with schizophrenia, with its incidence ranging from 10% to 20% during acute episodes of the disorder (5). Stupor significantly increases the burden of illness, impairs social functioning, and worsens prognosis, highlighting the need for timely recognition and effective intervention (6).

Pharmacological and somatic treatments have been widely applied in clinical settings. Benzodiazepines, particularly lorazepam, are widely used first-line treatments for stupor due to their effectiveness in rapidly relieving psychomotor inhibition (7, 8). Studies have found that its effective response rate can be as high as 75% (8). However, some patients may have refractory responses, and prolonged use can lead to dependence (9). Other therapeutic options, including N-methyl-D-aspartate (NMDA) receptor antagonists such as amantadine and memantine, may enhance efficacy when combined with benzodiazepines (10, 11). Electroconvulsive therapy (ECT) has also proven effective, particularly in acute or refractory stupor, and is often recommended for rapid clinical improvement (12). ECT is considered an evidence-based treatment for catatonia, with substantial support in the literature for its role in managing this condition (13–15).

In clinical practice, antipsychotic medications are frequently administered in the treatment of stupor, particularly when symptoms arise in the context of schizophrenia, schizoaffective disorder, or mood disorders with psychotic features (16, 17). patients often receive diverse antipsychotic regimens and dynamic dose adjustments during acute hospitalization, the total antipsychotic burden may influence treatment outcomes. Chlorpromazine, as a classic antipsychotic, is widely used for dose conversion and in numerous clinical studies (18, 19). By converting doses to chlorpromazine equivalents, comparisons between different medications can be made, helping to standardize treatment protocols. To better analyze the relationship between treatment response and drug dosage, this study chose to use chlorpromazine-equivalent dosing as the standardized dosage measure for antipsychotic medications.

Despite the availability of multiple treatment modalities, substantial inter-individual variability in therapeutic outcomes remains a major challenge. Early identification of predictors associated with treatment response may enable personalized treatment strategies, enhance clinical decision-making, and ultimately improve patient prognosis. Increasing evidence suggests that immune dysregulation and inflammatory processes may contribute to catatonia and related stuporous states. Subclinical systemic inflammation has been implicated by studies reporting elevated blood cell–based inflammatory ratios and associations between catatonia-like behaviors and peripheral inflammatory indices in patients with schizophrenia (20, 21). Moreover, complete blood count-derived inflammatory markers are increasingly used as accessible and cost-effective indicators of systemic inflammation in mood and psychotic disorders (22). On this basis, we included these peripheral inflammatory markers in the present study to explore whether they are associated with short-term treatment response in stupor. Nevertheless, reliable biomarkers or clinical predictors that can guide individualized treatment remain lacking. Therefore, this retrospective study aimed to explore clinical predictors of treatment efficacy in patients with stupor. By analyzing demographic, clinical, and therapeutic factors, we sought to identify variables associated with improved outcomes and provide evidence to guide individualized treatment planning.

## 2 Methods

### 2.1 Design and population

This retrospective study initially considered 45 patients diagnosed with stupor who were hospitalized at Shandong Daizhuang Hospital between January 2010 and August 2024. Inclusion criteria were as follows: (1) hospitalization at Shandong Daizhuang Hospital within the study period; (2) a diagnosis of stupor based on the International Classification of Diseases, 10th Revision (ICD-10), including catatonic stupor (F20.2), depressive stupor (F32.3), organic stupor (F06.1), or dissociative stupor (F44.2); (3) a clearly documented stupor episode with corresponding underlying psychiatric or medical diagnosis; (4) availability of complete demographic, clinical, medication, and peripheral blood data at admission; and (5) a recorded Clinical Global Impression–Improvement (CGI-I) rating at 2 weeks after admission. Exclusion criteria were: (1) incomplete key clinical or laboratory data; (2) comorbid severe psychiatric disorders that could substantially interfere with the assessment of stupor or treatment response; or (3) failure to complete the 2-week treatment process. According to these criteria, 5 patients were excluded (incomplete clinical data, n=3; comorbid severe psychiatric disorders, n=1; failure to complete the treatment process, n=1), and 40 patients were ultimately included in the final analysis.

The study was approved by the Ethics Committee of Shandong Daizhuang Hospital and conducted in accordance with the Declaration of Helsinki (2024-No.58-202410HM-1). Informed consent was not required for this study, as it was exempt from institutional review. The Chinese Clinical Trial Registry lists the clinical trial under the registration number ChiCTR2400093247.

### 2.2 Clinical assessments and data collection

Consistent with recent catatonia studies (23), treatment efficacy in our study was also assessed using the Clinical Global Impression–Improvement (CGI-I) scale at the end of two weeks of hospitalization. Previous research has shown that a CGI-I score of 3 reflects only minimal improvement and does not constitute a true clinical response (24, 25); therefore, based on the CGI-I ratings, patients were classified as responders (CGI-I scores of 1-2, indicating significant improvement) or non-responders (CGI-I scores of 3-4, indicating minimal or no improvement). Two authors (XZ and ZQ L) independently reviewed patient charts to determine efficacy responses in patients with stupor, resolving differences by consensus with experts (KL).

Demographic information collected included age, gender, marital status, educational level, occupation. Clinical characteristics included age of onset, and illness duration, and type of stupor (catatonic, depressive, organic, or dissociative).

Medication data were obtained from admission records and physician orders. Information included the use of antidepressants, mood stabilizers, benzodiazepines, and antipsychotics, as well as the administration of ECT. For quantitative analysis, psychotropic medication dosages were standardized using equivalent dose conversions. Antipsychotic dosages were converted to chlorpromazine equivalents (26), benzodiazepines to diazepam equivalents, and antidepressants to fluoxetine equivalents, following established conversion guidelines from prior literature (27). This approach allowed cross-comparison of different drugs within the same pharmacological class.

Peripheral blood parameters were collected on the second day after admission, including platelet count, monocytes, lymphocytes, neutrophils, eosinophils, and basophils. All participants underwent fasting venous blood collection (5 mL) after at least 8 hours of overnight fasting, using vacuum tubes containing dipotassium ethylenediaminetetraacetic acid (EDTA-K_2_) as an anticoagulant. The samples were promptly transported to the laboratory and analyzed immediately, with the hematology analyzer confirmed to be functioning properly and quality control values within the acceptable range prior to testing. Based on the test results, the following inflammatory indices were calculated: platelet-to-lymphocyte ratio (PLR), neutrophil-to-lymphocyte ratio (NLR), neutrophil-to-monocyte ratio (NMR), lymphocyte-to-monocyte ratio (LMR), and systemic immune-inflammation index (SII) (28). These hematological indices were used as surrogate markers to explore potential associations between immune-inflammatory status and treatment efficacy (29).

### 2.3 Statistical analyses

Data analyses were conducted using IBM SPSS Statistics version 27.0 (IBM Corp., Armonk, NY, USA). Descriptive statistics were calculated for demographic, clinical, medication, and inflammatory variables. Continuous variables were summarized as mean ± standard deviation (SD) when approximately normally distributed and as median with interquartile range (IQR) when skewed. Categorical variables were presented as counts and percentages. Between-group comparisons were performed using the independent-samples *t* test for normally distributed continuous variables and the Mann–Whitney *U* test for non-normally distributed variables, and the chi-square test or Fisher’s exact test for categorical variables, as appropriate. To evaluate the relationship between clinical factors and treatment response, univariate logistic regression analyses were conducted. Variables with statistical significance in univariate analysis were further entered into multivariate logistic regression models to identify independent predictors. Collinearity was assessed using variance inflation factors (VIFs). VIF diagnostics suggested substantial multicollinearity between ECT and chlorpromazine-equivalent dose; therefore, ECT was excluded from the final multivariable model to avoid unstable estimates and inflated standard errors. As a sensitivity analysis, we repeated the analyses after excluding patients who received ECT to examine the robustness of the associations in the non-ECT subgroup. To control for false positives due to multiple comparisons, we applied Bonferroni correction to adjust the p-values. Receiver operating characteristic (ROC) curves were generated on the entire dataset (n=40) to assess the predictive accuracy of significant variables. Because of the limited sample size, no training/test split or cross-validation was applied, and the ROC analysis should therefore be considered exploratory. The optimal cut-off value was determined using the Youden index. All tests were two-tailed, and statistical significance was set at *P* < 0.05.

## 3 Result

### 3.1 Demographic and clinical characteristics according to treatment response

Among the 40 patients included, 24 (60.00%) were classified as responders (CGI-I scores 1-2), while 16 (40.0%) were non-responders. Twenty-one patients (52.50%) were male and 19 (47.50%) were female, with a mean age of 33.07 ± 14.73 years. Eighteen patients (45.00%) were married, 11 (27.50%) were employed, and 11 (27.50%) had completed high school education or above.

The mean age of onset was 30.30 ± 14.23 years, and the mean illness duration was 26.06 ± 51.99 months. With respect to stupor subtypes, 18 patients (45.00%) had depressive stupor, 8 (20.00%) had catatonic stupor, 9 (22.50%) had dissociative stupor, and 5 (12.50%) had organic stupor. In terms of treatment, 23 patients (57.50%) received antidepressants, 12 (30.00%) received mood stabilizers, 16 (40.00%) received benzodiazepines, 32 (80.00%) received antipsychotics, and 20 (50.00%) underwent ECT. The median chlorpromazine equivalent dose was 100.000 (50.000, 250.000) mg, the median fluoxetine equivalent dose was 21.235 (0.000, 40.500) mg, and the median diazepam equivalent dose was 0.000 (0.000, 5.000) mg (**Table 1**).

**Table 1:** Demographic and clinical characteristics of patients with stupor by treatment response.

Characteristics	Response group (n = 24)	*95% CI*	Non-response group (n = 16)	*95% CI*	*T, Z or χ^2^*	*Df*	*P value*
Age	32.542 ± 14.151	26.674-38.410	33.875 ± 15.999	25.314-42.436	0.277^a^	38	0.789
Gender					0.067^c^	1	0.796
Male	11 (45.83%)		8 (50.00%)				
Female	13 (54.16%)		8 (50.00%)			1	
Marital Status					0.269^c^		0.604
Married	10 (41.66%)		8 (50.00%)				
Single	14 (58.33%)		8 (50.00%)				
Occupation					2.302^c^	1	0.316
Farmers	5 (20.83%)		3 (18.75%)				
Staff	3 (12.50%)		0 (0.00%)				
Unemployed	16 (66.66%)		13 (81.25%)				
Education level					5.159^c^	1	0.161
Primary school	6 (25.00%)		8 (50.00%)				
Junior high school	12 (50.00%)		3 (18.75%)				
High school	4 (16.66%)		2 (12.50%)				
College	2 (8.33%)		3 (18.75%)				
Age of Onset	26.000 (18.000,45.500)	23.809-35.775	30.000 (16.500,42.750)	23.585-38.541	-0.290^b^	1	0.772
Illness Duration (Months)	2.000 (0.130,21.000)		27.759 ± 48.075		-0.940^b^	1	0.347
Stupor Classification					1.030^c^		0.794
Depressive Stupor	11 (45.83%)		7 (43.75%)				
Catatonic Stupor	5 (20.83%)		3 (18.75%)				
Dissociative Stupor	6 (25.00%)		3 (18.75%)				
Organic Stupor	2 (8.33%)		3 (18.75%)				
ECT					6.667^c^	1	**0.010**
Yes	16 (66.66%)		4 (25.00%)				
No	8 (33.33%)		12 (75.00%)				
Antidepressants					0.614^c^	1	0.433
Yes	15 (62.50%)		8 (50.00%)				
No	9 (37.50%)		8 (50.00%)				
Mood Stabilizer					0.040^c^	1	0.883
Yes	8 (33.33%)		4 (25.00%)				
No	16 (66.66%)		12 (75.00%)				
Benzodiazepines					0.851^c^	1	0.356
Yes	11 (45.83%)		5 (31.30%)				
No	13 (54.16%)		11 (68.80%)				
Antipsychotics					1.100^c^	1	0.229
Yes	21 (87.50%)		11 (68.75%)				
No	2 (12.50%)		5 (31.25%)				
Chlorpromazine Equivalent Dose (mg)	200.000 (100.000,341.500)	150.0-316.7	56.250 (0.000, 100.000)	0-100.0	3.418^b^	1	**0.001**
Diazepam Equivalent Dose (mg)	0.000 (0.000, 5.000)	0.0-3.8	0.000 (0.000, 5.000)	0.0-5.0	0.556^b^	1	0.578
Fluoxetine Equivalent Dose (mg)	27.690 (0.000, 55.125)	0.0-22.2	5.065 (0.000, 30.9)	0.0-23.5	1.338^b^	1	0.181

*T, Z*, or *χ²* values correspond to *t-*tests ^a^, *Mann–Whitney U* tests ^b^, or *chi-square* tests ^c^, respectively. *Df*, degrees of freedom.*95%CI,* 95% confidence interval. Bold values indicate statistical significance at *P < 0.05*. ECT, Electroconvulsive therapy.

There were no statistically significant differences between responders and non-responders with respect to age, sex, marital status, education level, employment status, age of onset, illness duration, or stupor subtypes (all *P* > 0.05). Peripheral inflammatory markers showed no significant group differences: PLR (*|Z|* = 0.028, *P* = 0.978), MLR (*|Z|* = 0.193, *P* = 0.847), NLR (*|Z|* = 0.539, *P* = 0.590), NMR (*t* = 0.295, P = 0.769), SII (*|Z|* = 0.580, *P* = 0.562) (see **Table 2**).

**Table 2:** Comparison of peripheral inflammatory markers between responders and non-responders.

Peripheral inflammatory markers	Response group (n = 24)	*95% CI*	Non-response group (n = 16)	*95% CI*	*|Z|*	*Df*	*P- value*
PLR	125.609 (105.741, 203.500)	106.667- 170.000	135.409 (113.667, 154.559)	115.000-160.000	0.028^b^	1	0.978
MLR	0.260 (0.175, 0.428)	0.222- 0.340	0.288 (0.191, 0.431)	0.200-0.373	0.193^b^	1	0.847
NLR	3.064 (1.524, 4.714)	2.000-4.050	2.971 (2.352, 3.883)	2.200-5.100	0.539^b^	1	0.590
NMR	10.463 ± 3.918	8.800-13.200	10.835 ± 3.883	8.200-13.300	0.295^a^	38	0.769
SII	560.811 (324.461, 1087.207)	480.000-8520.000	660.488 (507.647, 817.284)	520.000, 1091.000	0.580^b^	1	0.562

*T, Z*, or *χ²* values correspond to *t*-tests a, *Mann–Whitney U* tests b, or *chi-square* tests c, respectively. *Df*, degrees of freedom.*95%CI,* 95% confidence interval. PLR, platelet-to-lymphocyte ratio; MLR, monocyte-to-lymphocyte ratio; NLR, neutrophil-to-lymphocyte ratio; NMR, neutrophil-to-monocyte ratio; SII, systemic immune-inflammation index.

Most patients in both groups received antipsychotics, and the overall use of antipsychotics, antidepressants, mood stabilizers, and benzodiazepines did not differ significantly between responders and non-responders (all *P* > 0.05). However, the proportion of patients receiving ECT was significantly higher in the responder group than in the non-responder group (66.66% vs. 25.00%; *χ²* = 6.667, *P* = 0.010, Bonferroni corrected *P* = 0.150). In addition, responders had significantly higher chlorpromazine-equivalent doses than non-responders (*|Z|* = 3.418, *P* = 0.001, Bonferroni corrected *P* = 0.015). As shown in **Table 3**, olanzapine was more frequently prescribed in the responder group (*χ²* = 4.365, *P* = 0.037, Bonferroni corrected *P* = 0.296), whereas the use of other antipsychotic agents did not differ significantly between the two groups (**Table 3**).

**Table 3:** Comparison of antipsychotic medication use between responders and non-responders.

Antipsychotics	Response group (n = 24)	Non-response group (n = 16)	*χ^2^*	*Df*	*P-* value
Olanzapine	17 (70.80%)	6 (37.50%)	4.365	1	**0.037**
Quetiapine	8 (33.30%)	3 (18.75%)	1.024	1	0.312
Aripiprazole	2 (8.33%)	2 (12.50%)	0.185	1	0.667
Amisulpride	1 (4.16)	1 (6.25%)	0.088	1	0.767
Sulpiride	4 (16.66%)	1 (6.25%)	0.952	1	0.369
Risperidone	1 (4.16%)	0 (0.00%)	0.684	1	0.408
Clozapine	0 (0.00%)	1 (6.25%)	1.538	1	0.215
Paliperidone	0 (0.00%)	1 (6.25%)	1.538	1	0.215

*χ²* values correspond to *chi-square* tests. Bold values indicate statistical significance at *P* < *0.05. Df*, degrees of freedom.

### 3.2 Predictive factors associated with treatment efficacy in stupor

Univariate analysis showed that ECT (*OR* = 6.000, 95% *CI*:1.458–24.686, *P* = 0.013), olanzapine (*OR* = 4.048, 95% *CI*:1.058–15.478, *P =* 0.041), and the chlorpromazine equivalent dose (*OR* = 1.009, 95% *CI*:1.002–1.016, *P* = 0.015) were significantly associated with 14-day treatment response. However, due to the collinearity between ECT and antipsychotic doses, ECT was excluded from the final multivariate model. Thus, the final model after controlling for olanzapine use factors and excluding ECT showed that higher chlorpromazine equivalent dose was independently and positively correlated with treatment response (*OR* = 1.007, 95% *CI*: 0.998–1.017, *P* = 0.045). The Nagelkerke pseudo-R² for this model was 0.214, indicating a modest level of model fit. This suggests that the chlorpromazine-equivalent dose explains 21.4% of the variance in treatment response.

**Figure 1:**
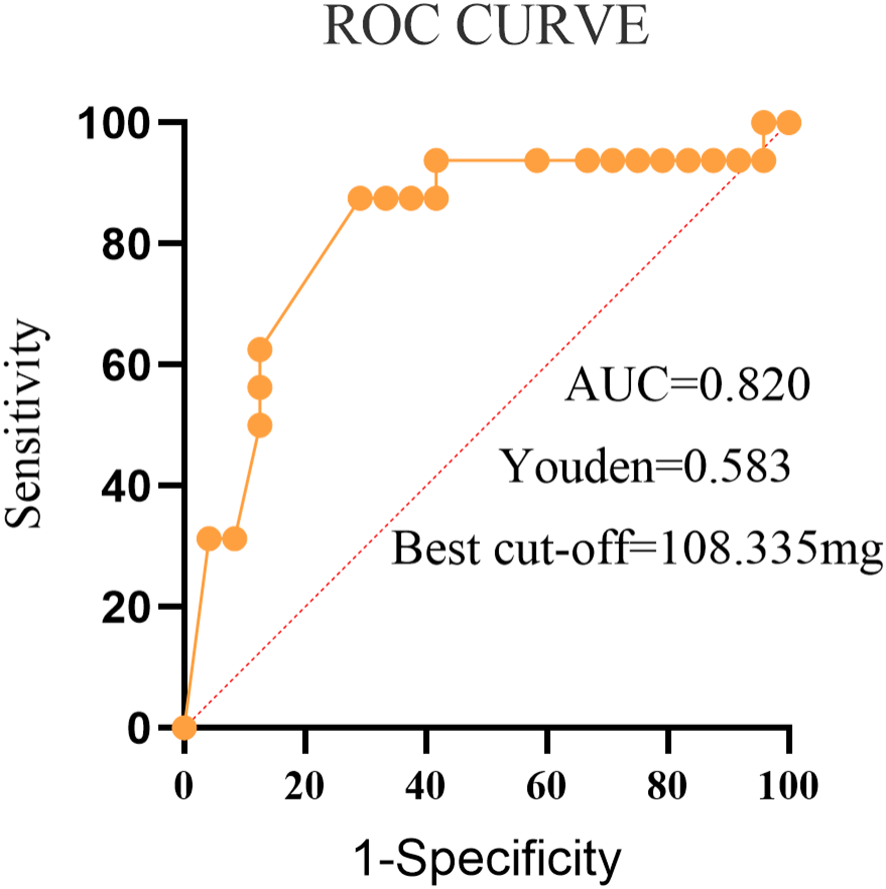
Receiver operating characteristic analysis of the chlorpromazine equivalent dose as the predictor of treatment efficacy in stupor.

ROC curve analysis showed that the chlorpromazine-equivalent dose had an AUC of 0.820 (95% *CI:* 0.677–0.963, *P* = 0.001). The sensitivity corresponding to the optimal cut-off value was 70.80%, the specificity was 87.50%, and the Youden index was 0.583. The optimal chlorpromazine-equivalent dose threshold for predicting treatment response at this time was approximately 108 mg (about 100–110 mg; point estimate: 108.335 mg), equivalent to approximately 4.3-5.4 mg of olanzapine (**Figure 1**) (statistically derived and exploratory, not a prescriptive clinical threshold).

## 4 Discussion

In this retrospective study of 40 patients with stupor, we identified several clinical predictors of treatment efficacy. Responders were more likely to receive higher chlorpromazine-equivalent doses and to be treated with olanzapine, and they were also more frequently treated with ECT. In multivariable logistic regression, the chlorpromazine-equivalent dose remained an independent predictor of response. Receiver operating characteristic analysis yielded an exploratory, data-driven cut-off of approximately 108 mg (about 100–110 mg) chlorpromazine-equivalent, corresponding to roughly 4.3-5.4 mg of olanzapine. This cut-off should be interpreted as exploratory rather than a prescriptive clinical threshold and should be validated in larger, prospective cohorts. By contrast, peripheral inflammatory markers did not differ significantly between responders and non-responders. Overall, these findings suggest that antipsychotic dosing and agent selection may contribute to treatment response, whereas the role of inflammation remains uncertain.

In this study, ROC analysis suggested an exploratory, data-driven predictive cut-off of approximately 108 mg (about 100–110 mg) chlorpromazine-equivalent, corresponding to roughly 4.3-5.4 mg of olanzapine. This estimate should not be interpreted as a prescriptive clinical threshold. Rather, it represents a statistically derived point associated with better outcomes and should be interpreted in light of known dose–response plateaus and the safety profile of antipsychotics in patients with stupor. These findings raise the possibility of a minimum effective antipsychotic dose in stupor, below which efficacy may be insufficient. They also suggest that, during the acute phase, appropriate dose escalation within recommended and evidence-based ranges may strengthen pharmacological effects and improve outcomes. This interpretation is consistent with prior studies supporting a dose-dependent effect of antipsychotics in stupor and suggests that careful upward titration may benefit treatment-resistant cases when safety limits are respected (30, 31). At the same time, dose escalation is unlikely to yield indefinite gains. Once antipsychotic exposure exceeds a certain level, further increases may provide little additional benefit while increasing the risk of adverse events. Such plateau effects may reflect receptor saturation, inter-individual variability, and pharmacokinetic constraints (32, 33). High-dose antipsychotic use also raises important safety concerns, particularly in vulnerable populations such as older adults and patients with substantial medical comorbidities. Prolonged exposure may promote desensitization, contribute to pharmacological tolerance and treatment resistance, and increase adverse drug reactions, potentially undermining subsequent therapeutic response in stupor (34, 35). Accordingly, any attempt to determine an “optimal” dose should balance efficacy against safety and be individualized with ongoing, comprehensive clinical monitoring to optimize outcomes. This study used chlorpromazine-equivalent dose as a standardized measure to minimize dose differences between antipsychotics, enabling effective comparisons across treatment regimens and providing valuable guidance for clinical decisions. While primarily focused on chlorpromazine equivalents, future research should explore dose effects across different antipsychotics for a more comprehensive assessment of treatment outcomes.

We also found no significant difference between the two groups in whether they used antipsychotic drugs or not. But there was a difference in the use of antipsychotic drugs between the two groups, specifically that the proportion of patients using olanzapine in the responding group (70.80%) was significantly higher than that in the non-responding group (37.50%), and univariate analysis showed that olanzapine use was significantly associated with 14-day efficacy. Together with the positive association between higher chlorpromazine-equivalent doses and improvement in stupor, this pattern is compatible with a dose-dependent antipsychotic effect and a possible specific contribution of olanzapine. Previous studies provide a plausible biological rationale for our findings. Olanzapine has been shown to modulate frontal and limbic activity during cognitive and emotional processing (36), and neuroimaging studies suggest that catatonia is characterized by dysfunction within these same front-subcortical circuits (37). Consistent with this, case reports describe catatonic symptoms improving with olanzapine treatment (33). Overall, our findings suggest that olanzapine may contribute to improvement in stupor, although no significant short-term efficacy difference was observed compared with other antipsychotics. This likely reflects methodological and power limitations rather than an absence of true between-drug differences. As a retrospective observational study, confounding by indication and other unmeasured variables cannot be excluded. Moreover, with a limited sample size and antipsychotics used largely within recommended dose ranges, short-term remission of stupor is probably driven by class effects, making subtle drug-specific differences difficult to detect. Prospective studies with adequate statistical power are needed to clarify the role of individual antipsychotics in catatonia and to determine whether olanzapine provides any meaningful therapeutic advantage.

Previous studies have shown that ECT is effective across a range of psychiatric disorders (38–41). It can enhance the release and transmission of key neurotransmitters (42), thereby improving affective disturbances and reducing inhibitory behaviors. Given that the motor inhibition observed in catatonia may similarly arise from neurotransmitter dysregulation within cortico-subcortical circuits (43), it is plausible that ECT exerts therapeutic benefits in this context as well. In our retrospective study, ECT was significantly associated with treatment response in the univariate analysis, beyond chlorpromazine-equivalent dose. However, this association was no longer significant in the multivariate analysis. This may be due to factors such as our small sample size, the short duration of observation, and the potential masking of ECT effects by concomitant medications. The American Psychiatric Association recommends ECT as a first-line treatment for patients with depressive stupor (44). We observed a higher incidence of depressive stupor in the response group, although we could not determine whether ECT was specifically more effective for this subtype. Nevertheless, our findings suggest that, in clinical practice, initiating ECT within the first two weeks of treatment may improve overall efficacy and alleviate stupor symptoms.

In this study, given the potential collinearity between ECT and antipsychotic dose, ECT was excluded from the final regression model to more accurately assess the independent effect of medication dose on treatment response. To examine the robustness of our findings, we further excluded patients who received ECT and conducted a sensitivity analysis in the remaining 20 patients without ECT, in which higher antipsychotic doses were not significantly associated with treatment response. This finding may be due to the reduced sample size and restricted dose variability after excluding patients treated with ECT, which diminished our ability to detect a dose–response relationship. Future studies with larger samples and a broader range of antipsychotic doses are warranted to more conclusively elucidate the relationship between antipsychotic dosage and treatment response. In addition, we performed chi-square tests comparing treatment efficacy across the four stupor subtypes and found no significant differences. Based on previous studies on stupor improvement (42, 45), we classified patients into four subtypes-catatonic, depressive, dissociative, and organic-and analyzed responders and non-responders within each subtype. The results suggest that stupor subtypes may modulate treatment response.

Firstly, our study found that in the catatonic group, there was a significant difference in ECT use, with more responders receiving ECT, but logistic regression did not confirm its independent predictive value, possibly due to small sample size or confounders. Secondly, in the depressive group, significant difference was found in ECT use, with responders receiving more ECT. However, regression analysis did not confirm an independent effect of ECT, indicating the need for further investigation. Thirdly, in the dissociative group, the use of olanzapine was significantly higher in responders, suggesting olanzapine may be more effective for this subtype. Finally, in the organic group, marital status differed significantly, with married patients showing better response, highlighting the potential importance of social context in treatment outcomes.

Previous studies suggest that stupor may be associated with immune inflammation. For example, patients with stupor have been reported to show higher inflammatory marker levels than healthy individuals, with C-reactive protein (CRP) significantly elevated during stupor (46). Recent research has also reported an association between catatonia-like behavior and inflammatory markers in patients with schizophrenia, suggesting a crosstalk between psychiatric symptoms and immune activation (18). Furthermore, meta-analytic evidence has demonstrated that NLR and PLR are significantly elevated in patients with mood disorders compared with healthy controls (47). In addition, an increased MLR has been reported in both schizophrenia and bipolar disorder, further supporting the notion that peripheral inflammatory markers may have transdiagnostic relevance across psychiatric conditions (48). Despite these findings, we did not observe significant group differences in peripheral blood indices. This discrepancy may reflect variability in patient characteristics, prior use of anti-inflammatory medications, and the limited sensitivity of routine hematological measures (49, 50). Importantly, routine CBC-derived indices (e.g., NLR, PLR, and MLR) are indirect and nonspecific proxies of inflammation and can be influenced by a range of factors such as acute stress response, infection, hydration status, smoking, metabolic conditions, and concomitant medications, which may limit their interpretability in relation to stupor-specific immune mechanisms. Retrospective studies inherently have certain limitations, particularly in terms of data collection, patient selection, and potential interference from prior treatments. First, as this study is based on historical data, it was not possible to uniformly control for whether patients had received anti-inflammatory or immune-modulating treatments prior to treatment, which may have led to bias in the levels of inflammatory markers. Additionally, immune processes relevant to stupor may be dynamic, and a single time-point assessment on day 2 after admission may be insufficient to capture temporal fluctuations or critical windows of immune activation associated with stupor onset, persistence, or resolution, potentially affecting the predictive ability of inflammatory markers for treatment outcomes. Although we did not find a predictive role for inflammatory markers in this study, this does not rule out the potential role of inflammation in treatment response. Future prospective studies with more rigorous designs are needed to further investigate the role of inflammatory responses in treatment outcomes.

This study suggests that optimizing antipsychotic dosage, considering olanzapine use, and integrating ECT may improve treatment outcomes in stupor. The results provide practical guidance for early clinical decision-making. However, several limitations should be acknowledged. First, the retrospective design and relatively small sample size (n = 40) may limit generalizability and reduce statistical power, particularly for detecting small-to-moderate effects of inflammatory markers or specific pharmacological strategies. Nevertheless, the sample size is broadly comparable to that of previous clinical studies of stupor or catatonia, which have often relied on small case series because of the low incidence and clinical complexity of these conditions. Second, we only assessed treatment response at two weeks, which is insufficient to evaluate sustained remission or relapse risk. Future studies should include long-term follow-up to better understand the durability of treatment effects and recurrence potential. Third, the lack of detailed inflammatory profiling limits the ability to draw firm conclusions regarding immune contributions. Fourth, the ROC analysis was conducted on the same dataset that was used to fit the logistic regression model, without an independent validation cohort or cross-validation because of the small sample size, which may lead to an overestimation of the predictive performance of the identified chlorpromazine equivalent dose. Prospective, large-scale studies with longitudinal follow-up are warranted to validate and extend these findings.

## 5 Conclusion

This retrospective study found that higher chlorpromazine-equivalent doses and the use of olanzapine were associated with better treatment response in patients with stupor, with a data-driven, exploratory optimal threshold of approximately 108 mg (about 100–110 mg; point estimate: 108.335 mg) (roughly 4.3-5.4 mg of olanzapine). ECT may further enhance early therapeutic outcomes. In contrast, peripheral inflammatory markers showed no significant differences between responders and non-responders, suggesting limited predictive value. Future studies with larger cohorts and more sensitive biomarkers are needed to clarify the potential role of inflammation in stupor.
